# Non-occlusive Mesenteric Ischemia Following Transcatheter Aortic Valve Replacement: A Case Report

**DOI:** 10.7759/cureus.87468

**Published:** 2025-07-07

**Authors:** George C Michalopoulos, Viktoriya Bikeyeva, Giovanni Paolella, Joseph Adams, Andrii Labchuk, Adib Chaus

**Affiliations:** 1 Internal Medicine, Advocate Lutheran General Hospital, Park Ridge, USA; 2 Cardiology, Advocate Lutheran General Hospital, Park Ridge, USA

**Keywords:** interventional cardiology corner, non-occlusive mesenteric ischemia, pre-operative evaluation, procedural complications, transcatheter aortic valve replacement

## Abstract

Non-occlusive mesenteric ischemia (NOMI) is a rare complication after transcatheter aortic valve replacement (TAVR), with a poorly understood pathogenesis. We present the case of a 79-year-old female with a history of surgically repaired abdominal aortic aneurysm (AAA) and stable thoracic aortic aneurysm (TAA) who developed NOMI after TAVR, resulting in extensive bowel necrosis and patient mortality. Our case highlights the special attention that must be paid to patients with a history of endovascular interventions prior to TAVR due to the risk of postprocedural NOMI. We propose that NOMI after TAVR occurs from the reduction in cardiac output due to rapid pacing during valve deployment, resulting in worsening splanchnic hypoperfusion in patients with existing mesenteric vascular disease.

## Introduction

Aortic stenosis in recent decades has become the predominant form of valvular heart disease in developed countries worldwide, with degenerative calcification accounting for 80% of these cases [1]. Severe aortic stenosis is defined by a peak velocity greater than 4 meters per second (m/sec), aortic valve area less than 1 cm^2^ (or <0.6 cm^2^/m^2^ adjusting for body surface area), and a mean pressure gradient greater than 40 mmHg [2]. Severe aortic stenosis can be further defined as low-flow-low gradient when the transvalvular stroke volume index is less than 35 mL/m^2^ and left ventricle ejection fraction (EF) is less than 40% or paradoxical low-flow-low gradient when EF is greater than 50% [2]. Low-flow-low-gradient aortic stenosis accounts for approximately 25% of all total cases of aortic stenosis [1]. Patients with low-flow-low-gradient severe aortic stenosis often have symptoms refractory to medical management, with an estimated three-year survival rate of less than 50% without procedural intervention [2]. The mainstay of treatment for symptomatic low-flow-low-gradient severe aortic stenosis involves interventions such as transcatheter aortic valve replacement (TAVR) or surgical aortic valve replacement (SAVR), based upon the patient’s surgical candidacy [1]. Patients with severe low-flow-low gradient aortic stenosis who undergo TAVR were shown to have a greater two-year average survival of 76% compared to 46% in patients who were only managed medically [3].

Since 2019, the volume of TAVR performed annually has surpassed SAVR because of its favorable outcomes and reduction in median length of hospital stay [4]. Well-reported potential complications of TAVR include high-grade atrioventricular (AV) block due to the close proximity of the aortic annulus to the bundle of His and left bundle branch, cardiac tamponade due to ventricular perforation or aortic annulus rupture, and vessel injury at the access site [5]. Non-occlusive mesenteric ischemia (NOMI) is a lesser-known and rare complication after TAVR with high patient mortality and a poorly understood multifactorial pathogenesis. We propose that rapid pacing during valve deployment results in a reduction in cardiac output, leading to worsening splanchnic hypoperfusion in patients with known mesenteric vascular disease.

Given the increasing volume of TAVR performed each year, it is imperative to raise awareness of this potential complication in order to identify effective pre-procedural risk stratification methods, post-procedure assessments, and effective interventions.

This article was part of a poster presentation given at the 2025 Advocate Aurora Health Annual Scientific Day on May 21, 2025.

## Case presentation

**Figure 1:**
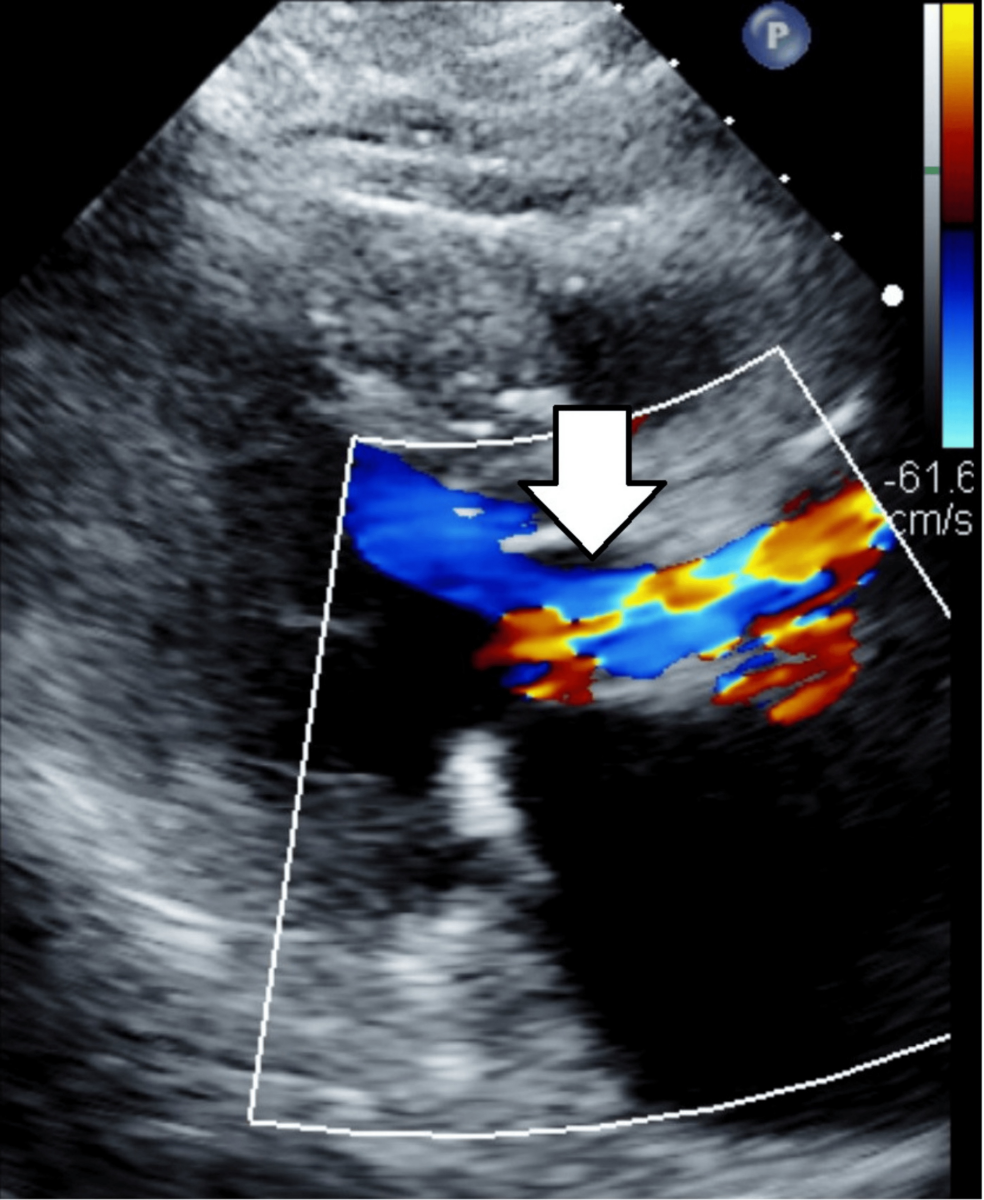
Pre-procedure Transthoracic Echocardiogram Low-flow, low-gradient aortic stenosis, as indicated by the arrow. Aortic valve area (AVA) = 0.7 cm^2^, mean gradient = 36 mmHg, aortic velocity = 3.0 m/sec, left ventricular outflow tract to aortic valve (LVOT/AV) ratio = 0.26, left ventricular ejection fraction (LVEF) = 45%, and transvalvular stroke volume index = 32 mL/m^2^.

The patient is a 79-year-old female with a history of abdominal aortic aneurysm (AAA) status post endovascular aneurysm repair (EVAR) and stable thoracic aortic aneurysm (TAA), being evaluated for recurrent heart failure exacerbations and progressive dyspnea. Transthoracic echocardiogram (TTE) demonstrated low-flow-low-gradient aortic stenosis with EF of 45%, transvalvular stroke volume index of 32 mL/m^2^, left ventricle end diastolic diameter adjusted for body surface area of 3.6 cm/m^2^, and normal appearing myocardium (Figure 1 and Video 1).

**Video 1:** Pre-procedure Transthoracic Echocardiogram This video demonstrates low-flow, low-gradient aortic stenosis. Aortic valve area (AVA) = 0.7 cm^2^, mean gradient = 36 mmHg, aortic velocity = 3.0 m/sec, left ventricular outflow tract to aortic valve (LVOT/AV) ratio = 0.26, left ventricular ejection fraction (LVEF) = 45%, and transvalvular stroke volume index = 32 mL/m^2^.

**Figure 2:**
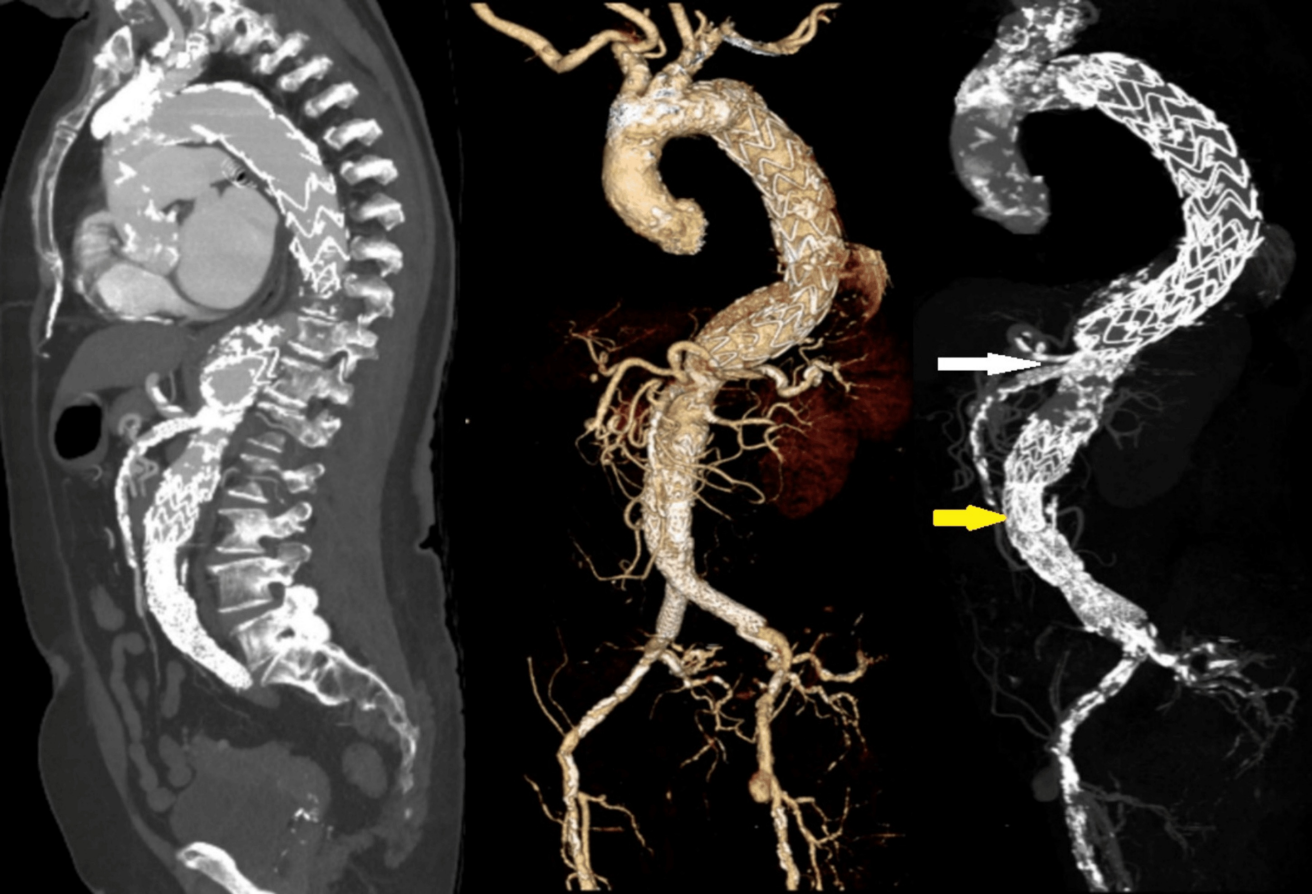
Pre-procedure Computed Tomography Angiography (CTA) of the Chest, Abdomen, and Pelvis The left panel shows a sagittal view of the thoracic and abdominal vasculature. The middle and right panels show the thoracic and abdominal vasculature isolated from surrounding organs and structures. All panels demonstrate a patent descending thoracic aorta, superior mesenteric artery (SMA), and infra-renal aortic stent graft (yellow arrow). Celiac ostium stenosis is re-demonstrated (white arrow), along with extensive abdominal aortic and mesenteric vascular calcification.

Given her recurrent heart failure exacerbations and symptoms refractory to medical management, she elected to pursue evaluation for aortic valve replacement. The patient’s Society of Thoracic Surgeons (STS) score for short-term/operative risk was calculated to be 4.21% for operative mortality and 14.4% for perioperative morbidity and mortality. The patient was deemed intermediate risk for SAVR, and instead, she elected to proceed with TAVR. Computed tomography (CT) performed as part of the preprocedural planning confirmed stable grafts, TAA, and chronic non-obstructive stenosis of the celiac, superior, and inferior mesenteric arteries with adequate appearing collateral circulation (Figure 2).

**Figure 3:**
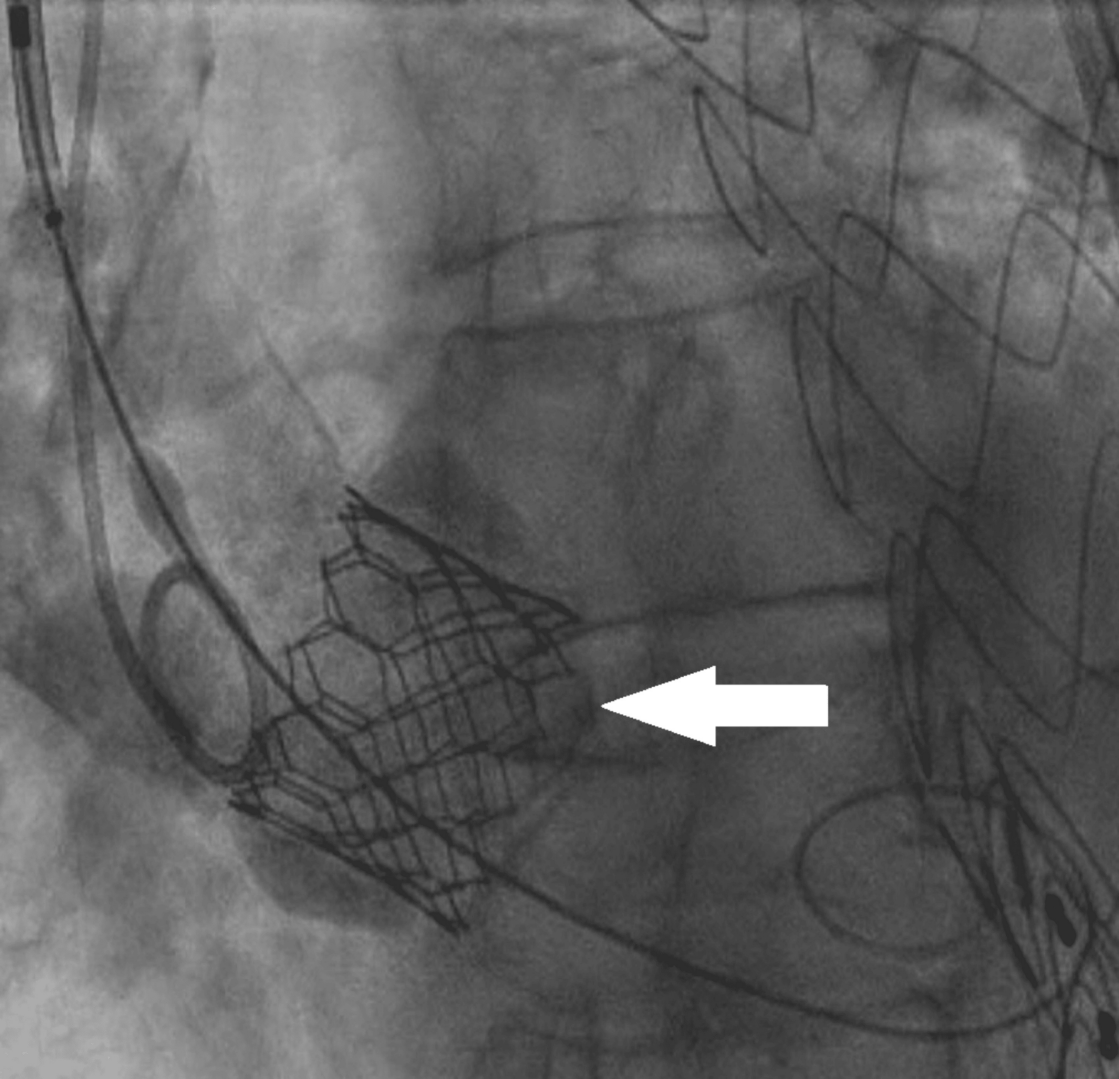
Intra-procedure Fluoroscopic Aortography Fluoroscopic aortography showing successful deployment of a 26 mm SAPIEN Ultra Resilia aortic valve (white arrow).

Right femoral and right radial arterial access was obtained without difficulty using a micropuncture needle with the Seldinger technique and 6-French sheaths. A 5-French pigtail catheter was positioned in the ascending aorta without difficulty or encountered resistance from previous aortic grafts. The right femoral arterial sheath was then exchanged for a 14 French Edwards E-sheath (Edwards Lifesciences, Irvine, CA, USA) using the Seldinger technique. A SAPIEN Ultra Resilia 26 mm valve (Edwards Lifesciences) was successfully implanted without difficulty and without evidence of paravalvular leak or dissection on angiography (Figure 3). The patient was hypertensive with a systolic blood pressure of 148 mmHg and diastolic pressure of 84 mmHg prior to the procedure, and there were no reported episodes of hypotension during the procedure. The patient was rapidly paced for 20 seconds during valve deployment. A temporary pacemaker was placed due to transient AV block. The patient was admitted to the medical intensive care unit for post-procedure monitoring. Blood pressure at that time was 157 mmHg systolic and 75 mmHg diastolic.

**Figure 4:**
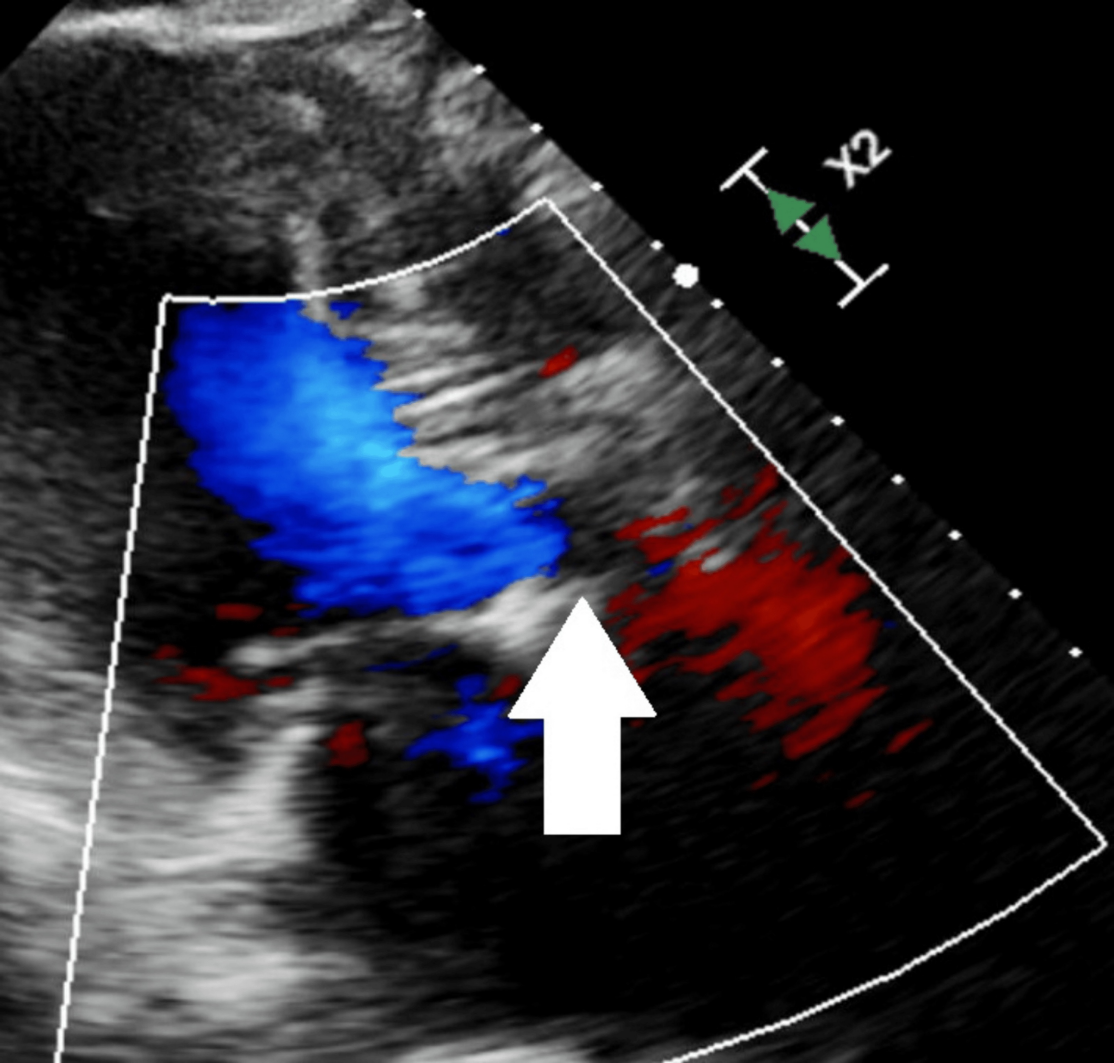
Post-procedure Transthoracic Echocardiogram (TTE) Post-procedural TTE demonstrating a well-positioned 26 mm SAPIEN Ultra Resilia valve (white arrow), with improved parameters: aortic valve gradient = 11 mmHg, LVOT/AV ratio = 0.53, velocity = 1.7 m/sec, LVEF = 65%, and transvalvular stroke volume index = 41 mL/m^2^. LVOT: left ventricular outflow tract; AV: aortic valve; LVEF: left ventricular ejection fraction

TTE performed post-procedure revealed an improved aortic valve gradient and velocity, a transvalvular stroke volume index of 41 mL/m^2^, and an increased EF of 65% (Figure 4 and Video 2).

**Video 2:** Post-procedure Transthoracic Echocardiogram (TTE) Post-procedure TTE demonstrates a well-positioned 26 mm SAPIEN Ultra Resilia valve, with improved aortic valve gradient (11 mmHg), LVOT/AV ratio (0.53), velocity (1.7 m/sec), and left ventricular ejection fraction (LVEF = 65%). LVOT: left ventricular outflow tract; AV: aortic valve

**Figure 5:**
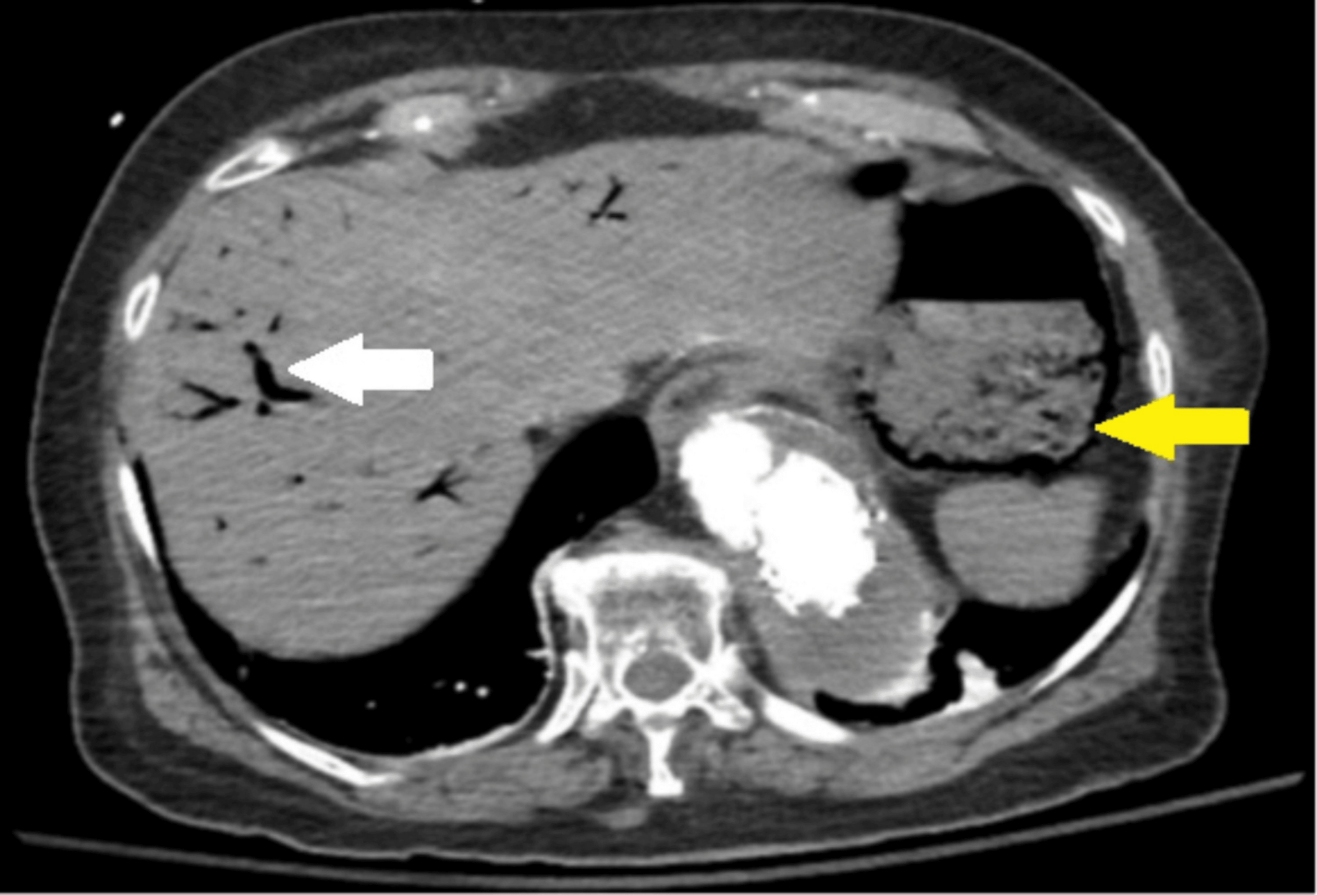
Computed Tomography Angiography (CTA) of the Abdomen and Pelvis CTA demonstrates acute gastric pneumatosis (yellow arrow) and hepatic pneumatosis (white arrow).

**Figure 6:**
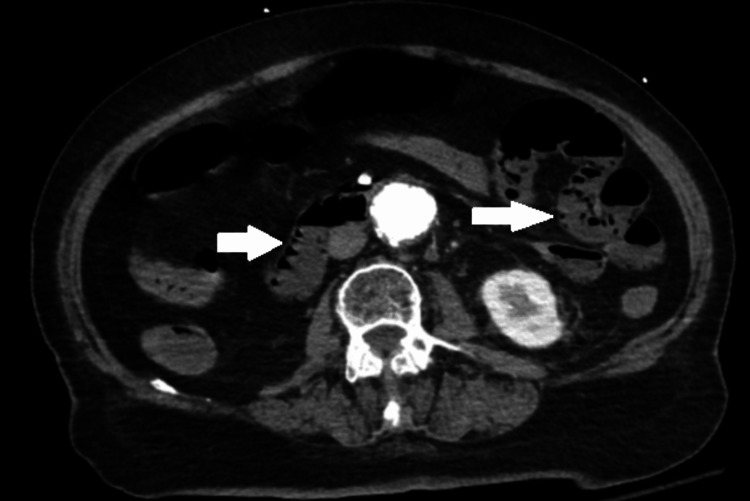
Computed Tomography (CT) of the Abdomen and Pelvis CT demonstrates acute duodenal and mesenteric pneumatosis (white arrows), suspicious for acute mesenteric ischemia with associated bowel necrosis.

Twenty-four hours later, the patient developed profuse diarrhea and severe left-sided abdominal pain. Labs at that time demonstrated a white blood cell count (WBC) of 18.2 K/mcL, with neutrophilic predominance and lactic acid of 2.4 mmol/L. The patient was started on empiric piperacillin-tazobactam for suspected intra-abdominal infection, and fluid resuscitation was performed with good response, forgoing the need to initiate vasopressor support. Stool samples for *Clostridium difficile*, GI pathogen panel, methicillin-resistant *Staphylococcus aureus* (MRSA) screening, and blood cultures were collected, all of which returned negative results. A STAT mesenteric CT angiography (CTA) performed at that time (approximately 25 hours post-procedure) showed extensive intrahepatic, gastric, and mesenteric pneumatosis (Figures 5, 6).

General and vascular surgical services were consulted and recommended emergent laparotomy. Emergent laparotomy was performed at 26 hours post-procedure, and confirmed pale tissue with associated necrosis extending from the gastroesophageal junction to the distal small bowel. No intraoperative specimens or tissue biopsies were collected due to the frankly ischemic appearance of the bowels. Given the extent of necrosis with overall poor prognosis, the decision was made to forgo surgical resection and instead proceed with palliative treatment.

Post-laparotomy, the patient’s lactic acid level rose to 4.6 mmol/L, and WBC increased to 19.2 K/mcL. The patient became progressively hypotensive with mean arterial pressures below 65 mmHg, necessitating the initiation of norepinephrine infusion at 20 mcg/minute. At this time, a discussion was had with the patient's family, who decided that they would like no further escalation of medical treatments or interventions. The patient was started on a continuous morphine infusion and expired approximately 36 hours post-procedure.

## Discussion

This case is intended to raise awareness of this rare but often fatal complication of TAVR in order to allow for earlier recognition and possible intervention when NOMI develops. Learning points from this case include recognizing high-risk patient characteristics prior to TAVR, the potential benefit of serial abdominal examinations and lactic acid trends for post-procedure monitoring, and speculative treatments for NOMI.

Our patient had multiple risk factors, such as prior endovascular interventions, reduced EF, and known non-obstructive stenosis of the celiac arteries with high-grade stenosis of the superior and inferior mesenteric arteries. These factors all likely contributed to the increased risk and potential predisposition to developing NOMI. Patients with extensive mesenteric vascular disease have diminished perfusion at baseline with minimal collaterals available to compensate, making them very sensitive to reductions in perfusion and at greater risk for NOMI. Rapid pacing during TAVR deployment likely led to mesenteric ischemia from reduced cardiac output, causing acute worsening of chronic splanchnic hypoperfusion despite apparently adequate collateral circulation on previous imaging studies.

Differentials also considered included thromboembolic occlusion, vasospasm of the mesenteric vasculature, and acute infectious etiology. Thromboembolic occlusion from underlying arrhythmia was unlikely since the patient had no prior history or current evidence of atrial fibrillation. Transesophageal echocardiography was not utilized prior to or during the TAVR procedure, but the possibility of an unidentified left atrial thrombus was unlikely. Thromboembolic occlusion from instrumentation in the abdominal or thoracic aorta was considered; however, there was no difficulty encountered when passing instruments through these vessels, and they were widely patent on pre-procedure CTA. Thromboembolic occlusion from implantation of the prosthetic aortic valve was considered but was less likely given the ease of implantation and no need for additional or excessive instrumentation of the aortic valve. Acute infectious etiology was initially suspected given the patient's acute leukocytosis, diarrhea, lactic acidosis, and need for fluid resuscitation. Infectious etiology was later refuted given the negative infectious evaluation and absence of fever.

Special attention must be paid to patients with a history of endovascular interventions such as EVAR or thoracic endovascular aortic repair (TEVAR), particularly in regard to the pre-procedural assessment of the mesenteric vasculature. Imaging modalities such as mesenteric CTA or magnetic resonance angiography (MRA) are the preferred non-invasive methods of assessing the mesenteric vasculature, with CTA demonstrating better assessment of the visceral branches of the mesenteric vessels [6]. Utilizing these imaging modalities for pre-procedure assessment could afford further risk stratification and prevention of NOMI by precluding patients who demonstrate extensive mesenteric vascular disease. Additional investigation must be performed to evaluate the efficacy of these imaging modalities used for this purpose and establish a validated risk assessment protocol. Mesenteric ischemia can occur unpredictably; thus, even with advanced imaging and establishment of a validated risk assessment protocol, the risk can never be completely eliminated.

Symptoms of NOMI often present late, after extensive ischemia has developed. Post-procedural monitoring of lactic acid trends and interval abdominal examinations may afford earlier detection and intervention.

Noguchi et al. described a similar case of NOMI where rapid pacing was used during valve deployment in a patient with stenotic iliac arteries [7]. Thirty hours after TAVR, the patient developed severe abdominal pain and lactic acidosis. An emergency angiogram was performed, which demonstrated vasospasm of the SMA. Intra-arterial administration of prostaglandin E1 alleviated the acute vasospasm, and a continuous infusion of papaverine was started. After two days, there was no evidence of mesenteric ischemia, and the infusion was safely discontinued [7].

Vasospasm as the etiology of NOMI is suspected to occur as a result of rapid pacing, potentially as a result of the increased sympathetic nervous system response to an acute reduction in cardiac output. Fluoroscopic angiography of the mesenteric vessels can be used to detect vasospasm as well as identify acute occlusion of the mesenteric vasculature. Vasospasm was also considered as a differential diagnosis in our patient’s case; however, given the acuity of their illness, late presentation, and evidence of extensive necrosis on CTA, mesenteric angiography was deferred, and emergent laparotomy was attempted to resect the necrotic bowel.

If the etiology of NOMI in our patient was confirmed to be from vasospasm of the mesenteric vasculature via fluoroscopic angiography, administration of intra-arterial vasodilators during the procedure may have acutely alleviated the vasospasm, and post-procedure intravenous vasodilators could have been used to maintain vessel relaxation. This treatment is largely speculative; however, if initiated immediately at the onset of the patient’s symptoms, it may provide significant benefit.

Current evidence of NOMI treatment is speculative and limited to several case reports. Further investigation, through meta-analysis or large studies, is needed to determine if systemic vasodilators are indeed effective in treating NOMI induced by vasospasm. Further investigation should also be performed to identify any alternative treatments to vasodilators and compare each of their respective mortality benefits to determine the most effective intervention.

## Conclusions

Patients with high-risk features such as prior EVAR or TEVAR, extensive mesenteric vascular disease, and low EF require careful consideration prior to TAVR because of the potential for NOMI. Rapid pacing during valve deployment can potentially cause mesenteric ischemia due to the acute reduction in cardiac output or from vasospasm of the mesenteric vasculature. Pre-procedure imaging modalities such as CTA or MRA may allow for additional risk stratification and assessment of the mesenteric vasculature prior to procedure, which could exclude patients considered to be high risk for post-procedure NOMI. Further investigation is needed to determine the efficacy of these modalities and their potential contribution to the development of a pre-procedure risk assessment protocol. Post-procedure monitoring protocols, such as trending lactic acid and interval abdominal examinations, may afford earlier detection of NOMI and subsequent intervention. Post-procedural monitoring with interval abdominal examinations may be limited by the number of available staff in each institution, given that these patients may not always be admitted to units that would allow for such close monitoring. Serial lactic acid trends may afford for earlier detection; however, it is not exclusively limited to NOMI, thus it can lead to false positives and increase the overall costs of care.

Early diagnosis and intervention for NOMI are critical because of the rapid clinical deterioration and high patient mortality rate. If vasospastic etiology is suspected, early involvement of vascular surgery or interventional radiology is recommended so fluoroscopic angiography of the mesenteric vasculature can be performed to confirm and administer intra-arterial vasodilators. Intravenous vasodilators administered after acute fluoroscopic angiographic intervention may be effective in maintaining vessel relaxation in instances of vasospasm. These interventions should be performed before the patient develops hemodynamic instability because the administration of systemic vasodilators may not be a viable option in those instances. These potential treatments are based on limited evidence, and further investigation is needed to evaluate their mortality benefits and identify additional effective interventions.
